# Immunization with an immunodominant self-peptide derived from glucose-6-phosphate isomerase induces arthritis in DBA/1 mice

**DOI:** 10.1186/ar2777

**Published:** 2009-07-29

**Authors:** Lisa Bruns, Oliver Frey, Lars Morawietz, Christiane Landgraf, Rudolf Volkmer, Thomas Kamradt

**Affiliations:** 1 Universitätsklinikum Jena, Institut für Immunologie, Leutragraben 3, Jena 07740, Germany; 2 Charité Universitätsmedizin Berlin, Institut für Pathologie, Charitéplatz 1, Berlin 10117, Germany; 3 Charité Universitätsmedizin Berlin, Institut für Medizinische Immunologie, Charitéplatz 1, Berlin 10117, Germany

## Abstract

**Introduction:**

T-helper (Th) lymphocytes are critically required for the pathogenesis of glucose-6-phosphate isomerase (G6PI)-induced arthritis, but neither the G6PI epitopes recognized by arthritogenic T cells nor their pathogenic effector functions have been fully elucidated to date. We aimed at identifying arthritogenic G6PI peptides.

**Methods:**

We used a library of overlapping peptides spanning the entire G6PI sequence to identify the epitopes recognized by G6PI-specific Th cells. Immunodominant peptides were then used to immunize mice. Arthritis development was evaluated clinically and histologically. The humoral and cellular immune responses upon peptide immunization were analyzed by ELISA and multiparameter flow cytometry, respectively.

**Results:**

We identified six immunodominant T-cell epitopes in DBA/1 mice, of which three are arthritogenic. One of these peptides (G6PI_469–483_) is identical in man and mice. Immunization with this peptide induces arthritis, which is less severe and of shorter duration than arthritis induced by immunization with full-length G6PI. Upon immunization with either G6PI or peptide, the antigen-specific Th cells produce IL-17, RANKL, IFNγ and TNFα.

**Conclusions:**

We identified immunodominant and arthritogenic epitopes of G6PI. Not all immunodominant peptides are arthritogenic. This is the first description of arthritis induced by immunization with a self-peptide in mice.

## Introduction

Autoreactive CD4^+ ^T-helper (Th) lymphocytes play a central role in the pathogenesis of autoimmune diseases [1]. Key to the development of immune responses is the binding of T-cell receptors on CD4^+ ^Th cells to their cognate peptide/MHC complex on the surface of antigen-presenting cells (APC). Among the well-established genetic risk factors for rheumatoid arthritis, *HLA-DRB1*, *PTPN22 *and *STAT4 *are relevant for T-cell function [2-4]. T cells are present in the inflamed synovial compartment [5,6]. These findings strongly suggest the concept that rheumatoid arthritis is Th-cell dependent, and that the associated HLA-DR molecules present peptides to autoreactive Th cells, which initiate the inflammatory process that ultimately leads to rheumatoid arthritis. This assumption is supported by the clinical benefits of treating rheumatoid arthritis patients with abatacept, a CTLA4–immunoglobulin fusion protein that blocks Th-cell costimulation, thus selectively inhibiting their activation [7,8]. Nevertheless, the specificity of the pathogenic Th cells in rheumatoid arthritis has been difficult to define.

In experimental animals, arthritis can be induced by systemic immunization with noncartilagenous antigens [9,10] or with cartilage-antigens including heterologous collagen type II [11], collagen type XI [12], cartilage oligomeric matrix protein [13] and proteoglycan [14] in complete Freund's adjuvant (CFA). The immune response of T cells to complex antigens is commonly focused on a small number of major epitopes. Although immunodominant collagen type II epitopes have been defined for different collagen-induced arthritis-susceptible strains of mice [11,15], and for proteoglycan [16], experimental arthritis cannot be induced by immunization with these immunodominant peptides [15,16]. In fact, even denatured collagen type II or its cyanobromide fragments are less efficient for arthritis induction than full-length, native collagen type II [17]. This lack of an identified arthritogenic epitope has been an obstacle to studying the role of Th cells in mouse models of arthritis. Collagen-induced arthritis is easily transferable with serum from arthritic animals or mixtures of monoclonal antibodies specific for collagen type II, reflecting a strong dependence on antibodies [18-20].

We recently described a model in which systemic immune responses to glucose-6-phosphate isomerase (G6PI) induce a peripheral symmetric polyarthritis in susceptible strains of mice [21,22]. In this model, arthritis development depends on T cells, B cells and innate immunity [21-25]. CD4^+ ^Th cells are crucial not only for the induction of the disease but also during the effector phase. Depletion of CD4^+ ^T cells in arthritic animals induces arthritis remission [21]. To understand better the role of Th cells in this model, we sought to determine the immunodominant epitopes in G6PI-induced arthritis. In the present article we describe the identification of six immunodominant G6PI epitopes and the induction of arthritis in DBA/1 mice by immunization with three of these peptides.

## Materials and methods

### Animals and arthritis induction

DBA/1 mice were bred and maintained under specific-pathogen free conditions in our animal facility. All animal experiments were approved by the Government Commission for Animal Protection (Registered Number 02-005/06).

Arthritis was induced in 6-week-old to 10-week-old DBA/1 mice by subcutaneous immunization at the base of the tail with either 400 μg recombinant human G6PI or 50 μg peptide in complete Freund's adjuvant (Sigma-Aldrich, Taufkirchen, Germany).

Clinical scores were determined daily for each paw independently, as previously described [21]. A score of 0 indicates no clinical signs of arthritis, 1 indicates slight swelling and redness, 2 indicates a strong swelling and redness, and 3 indicates massive swelling and redness. Arthritis incidence is almost 100% in this model, and the natural history is highly synchronized with arthritis onset on d9.

### Antibodies and reagents

The following mAbs were grown and purified from hybridoma supernatants in our laboratory: anti-CD16/CD32 (2.4G2) and anti-CD28 (37.51). Anti-IL-17A (eBio17B7)-Alexa 488, anti-TNFα (MP6-XT22)-Pacific Blue, anti-IFNγ (XMG1.2)-phycoerythrin-Cy7, anti-CD4 (RM4-5)-allophycocyanin-Alexa750 (APC-A750), anti-IL-2 (JES6-5H4)-fluorescein isothiocyanate, anti-IL-6 (MP5-20F3)-fluorescein isothiocyanate, anti-IL-10 (JES5-16E3)-APC, and anti-RANKL (IK22/5)-phycoerythrin were purchased from ebiosciences (San Diego, CA, USA). Anti-CD154 (MR1)-APC was purchased from Miltenyi Biotec (Bergisch Gladbach, Germany). Recombinant human G6PI was expressed in *Escherichia coli *BL21 as described previously [21].

### Peptides

Cellulose-bound peptides were prepared according to the standard SPOT synthesis protocol by a MultiPep SPOT-robot (INTAVIS Bioanalytical instruments AG, Köln, Germany) on a β-alanine-modified cellulose membrane as described elsewhere [26].

Each spot was eluted in 200 μl distilled H_2_O containing 5% dimethylsulfoxide, resulting in an approximate concentration of 350 to 650 μg/ml peptide solution. These peptide solutions were taken to create peptide pools resulting in a concentration of any single peptide of ~42 μg/ml. The final concentration for *in vitro *restimulation for every single peptide was ~1 μg/ml. Peptides for immunization were synthesized according to standard Fmoc machine protocols with the multiple peptide synthesizer SYRO II (MultiSynTec, Witten, Germany). The following peptides derived from human G6PI were synthesized: G6PI_65–79 _(MRMLVDLAKSRGVEA), G6PI_85–99 _(FNGEKINYTEGRAVL), G6PI_325–339 _(IWYINCFGCETHAML), G6PI_469–483 _(EGNRPTNSIVFTKLT), G6PI_497–511 _(KIFVQGIIWDINSFD) and G6PI_517–531 _(LGKQLAKKIEPELDG). Purity of the peptides was determined by high-performance liquid chromatography and the composition was monitored by matrix-assisted laser desorption/ionization time-of-flight mass spectroscopy.

### Histopathology

Microsections from mouse legs were prepared and stained with H & E as described previously [21]. Samples were viewed with a DMRBE microscope (Leitz, Wetzlar, Germany) by a pathologist who was blinded to the experimental setup. The severity was graded semiquantitatively in five steps from 0 (normal finding) to 4 (strong inflammation) as described previously [21,27].

### Proliferation assays

All cell cultures and assays were performed in RPMI 1640 supplemented with 10% FCS, 100 U/ml penicillin, 100 μg/ml streptomycin, and 50 μM 2-mercaptoethanol as described [21].

Cells were plated in a 96-well round-bottom plate (Greiner Bio-One, Solingen, Germany) at a density of 1 × 10^6 ^cells/ml culture medium. Cells were stimulated with either 10 μg/ml G6PI, 5 μl peptide pool or culture medium alone in triplicate for 72 hours. For the last 18 hours 1 μCi/well [^3^H]thymidine (GE Healthcare, München, Germany) was added. [^3^H]thymidine incorporation was measured with a β-scintillation counter. Results are displayed as the stimulation index, which is the quotient of the mean counts of cells that were stimulated and the mean count of cells cultured in medium alone. Results were considered positive if the stimulation index was at least 2 and the difference between the stimulated and the nonstimulated sample was more than 1,000 counts per minute.

### Flow cytometry

Single-cell suspensions from draining lymph nodes (inguinal, para-aortic, 1 × 10^7 ^cells/ml) were cultured in 48-well plates in the presence of 3 μg/ml anti-CD28 and either 20 μg/ml G6PI, 5 μg/ml peptide or medium alone for 6 hours. Brefeldin A (Sigma-Aldrich) was added to a final concentration of 5 μg/ml for the last 4 hours. Since CD154 upregulation occurs exclusively upon T-cell receptor signaling (and not upon bystander activation of T cells), possible lipopolysaccharide contamination of the antigen preparation does not influence this assay (data not shown). Cells were stained with a viability dye (Aqua fixable live/dead staining kit; Invitrogen, Karlsruhe, Germany) according to the manufacturer's instructions. After fixation with 2% paraformaldehyde for 20 minutes, cells were permeabilized with 0.5% saponin (Sigma-Aldrich) and incubated with anti-CD16/32 (2.4G2/75; 100 μg/ml) and rat IgG (200 μg/ml; Dianova, Hamburg, Germany) to prevent unspecific binding. Cells were stained for CD4, CD154 and cytokines. A seven-color panel was used to simultaneously analyze multiple cytokines on CD4^+^CD154^+ ^T cells using a BD LSR II flow cytometer (BD Biosciences, Heidelberg, Germany); 3,000,000 events were acquired for each sample.

Data were analyzed using FlowJo Software (TreeStar, Ashland, Oregon, USA). Doublets were excluded using forward scatter–area versus forward scatter–height parameters, followed by the selection of lymphocytes and live cells (Aqua-). Gates for CD154 were set using unstimulated control samples and gates for cytokine-positive cells were set using fluorescence-minus-one controls for the respective cytokine [28,29].

### Anti-G6PI-immunoglobulin ELISA

Titers of G6PI-specific antibodies were measured by ELISA as previously described [21]. Fourfold serial dilutions of the sera were incubated on G6PI-coated ELISA plates (Greiner, Frickenhausen, Germany) and bound immunoglobulins were detected with the mouse monoclonal isotyping kit (Sigma-Aldrich, Crailshaim, Germany). *o*-Phenylendiamine was used as the substrate, and the optical density was measured at 492 nm.

### Statistical analysis

All data are presented as the mean ± standard error of the mean unless otherwise indicated. Statistical analysis (non-parametric Mann–Whitney U test) was performed with SPSS 15.0 (SPSS Inc., Chicago, IL, USA).

## Results

### Mapping the immunodominant T-cell epitopes in G6PI-induced arthritis

**Figure 1:**
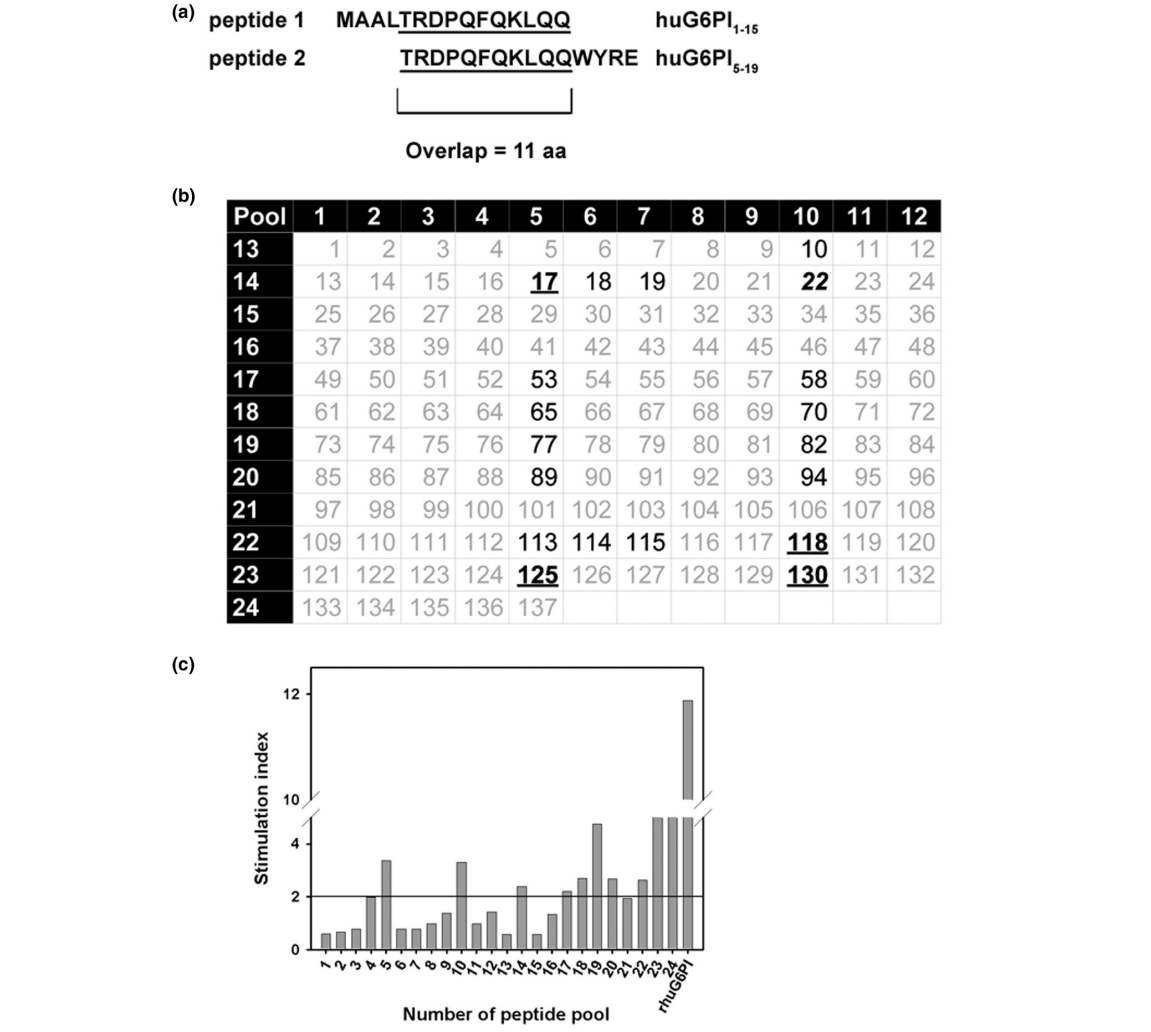
T-cell epitope mapping. **(a) **The peptide library covering the entire human glucose-6-phosphate-isomerase (G6PI) sequence contained 137 peptides of 15 amino acids length, overlapping by 11 amino acids (aa). **(b) **Epitope mapping with two-dimensional peptide pools. Pools are represented by horizontal lines or vertical columns (white numbers on black background). For example, Pool 14 contains peptides 13 through 24 (numbers on white background). Each peptide is represented in two pools. For example, peptide 22 is represented in Pools 10 and 14. For epitope mapping, DBA/1 mice were immunized with 100 μg G6PI in complete Freund's adjuvant (n = 3 per experiment). Single-cell suspensions were prepared from draining lymph nodes and the spleen 12 days after immunization, and proliferation assays were performed as described in Materials and methods. Three independent experiments were performed. The one peptide (peptide 22) that scored positive in all three experiments is highlighted in bold italic; peptides identified in two experiments are highlighted by bold underlined numerals (peptides 17, 118, 125 and 30); peptides identified in only one experiment are highlighted in bold and the other peptides are given in grey. **(c) **Results from one exemplary experiment are shown. Results are displayed as the stimulation index. A stimulation index >2 was considered positive.

To determine the immunodominant G6PI T-cell epitopes, we immunized DBA/1 mice with G6PI and examined T-cell proliferation in response to recombinant G6PI and a set of 137 overlapping 15mer peptides spanning the entire amino acid sequence of human G6PI (Figure 1a). For high-efficiency screening we designed 24 two-dimensional peptide pools such that each peptide was contained in two different pools (Figure 1b). Recombinant G6PI induced intensive T-cell proliferation (Figure 1c).

Peptide pools that induced a stimulation index ≥ 2 were considered to contain a T-cell epitope. Pools 10 and 14 induced stimulation indexes >2 in all three experiments performed. Therefore, peptide 22 (G6PI_85–99_), which is contained in both Pools 10 and 14 (Figure 1b), scored positive in all three experiments. Peptide 17 (G6PI_65–79_, contained in Pools 5 and 14), peptide 118 (G6PI_469–483_, contained in Pools 10 and 22), peptide 125 (G6PI_497–511_, contained in Pools 5 and 23) and peptide 130 (G6PI_517–531_, contained in Pools 10 and 23) scored positive in two out of three experiments (Figure 1b). In addition, several peptides yielded positive results in only one of the experiments (Figure 1b). Those five peptides that scored positive in at least two of the three experiments were chosen for further analysis.

While the present manuscript was in preparation, Iwanami and colleagues reported that G6PI_325–339 _was arthritogenic [30]. We therefore synthesized G6PI_325–339_, which is our peptide 82 and scored positive in one of three screening experiments, and performed additional experiments including this peptide.

### Immunization of DBA/1 mice with G6PI-derived peptides induces arthritis

**Figure 2:**
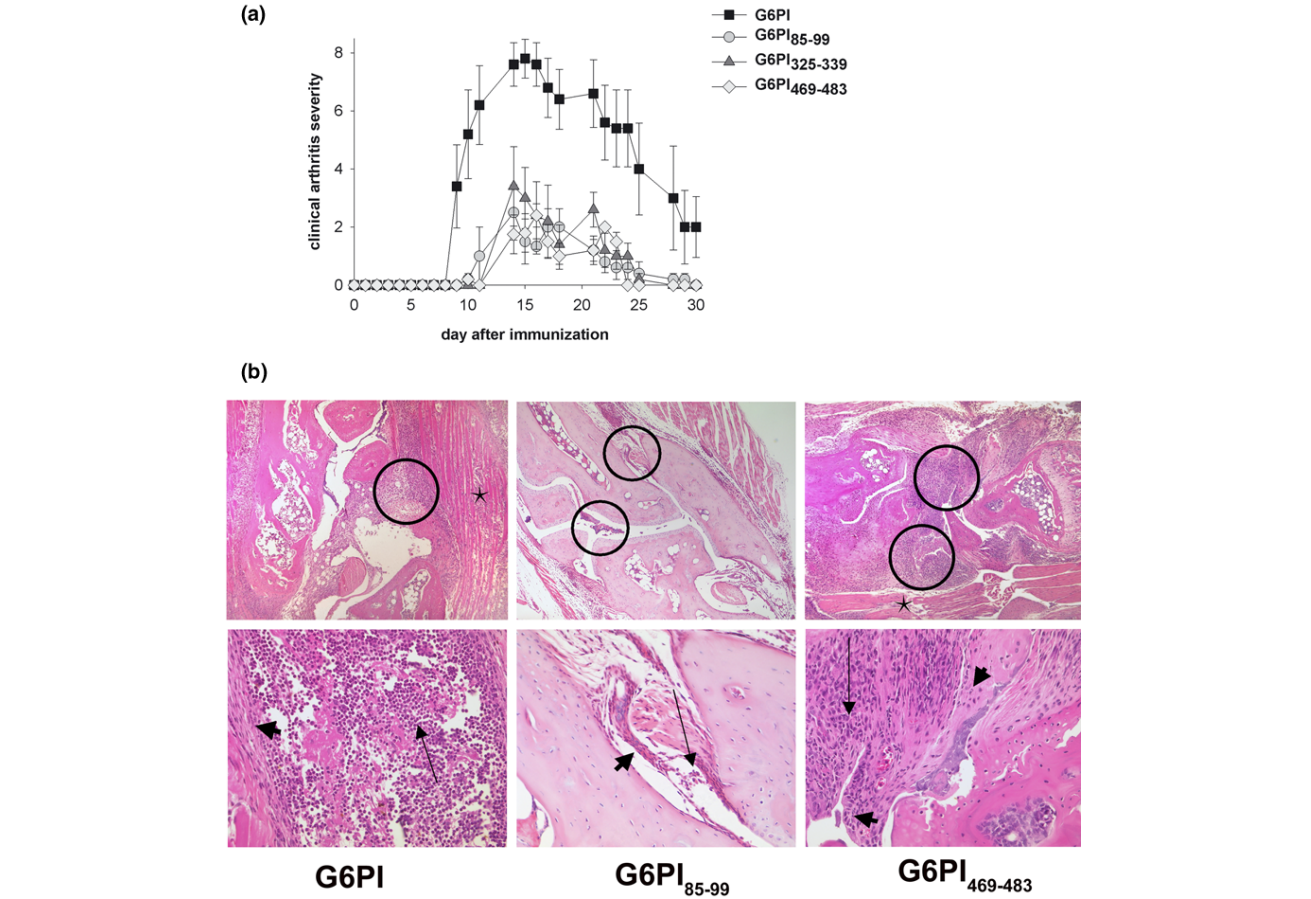
Immunization of DBA/1 mice with glucose-6-phosphate-isomerase-derived peptides induces arthritis. **(a) **Mice were immunized with either 400 μg glucose-6-phosphate-isomerase (G6PI), 50 μg G6PI_85–99_, 50 μg G6PI_325–339 _or 50 μg G6PI_469–483 _in complete Freund's adjuvant. Shown is the clinical severity of disease in arthritic mice in a representative experiment (n = 5 per group). Each peptide was tested in at least two independent experiments (Table 1). **(b) **The animals immunized with G6PI showed a strong synovialitis (circles) and inflammation of the adjacent skeletal muscle (star), which was dominated by neutrophilic granulocytes (small arrow) as a sign of acute inflammation and exhibited activation of synovial fibroblasts to a lesser extent (broad arrow). The synovial tissue was enlarged due to the dense inflammatory infiltrate; however, bone destruction was only focally observed. Similar but markedly less intense alterations could be observed in the animals immunized with G6PI_85–99_, whereas immunization with peptide G6PI_469–483 _resulted in an inflammation that was as strong as when using whole G6PI. (H & E; original magnification: upper row, 50×; lower row, 200×). Results show representative data from at least nine individual mice from two typical experiments.

Immunization of DBA/1 mice with full-length G6PI in CFA induces arthritis with a high incidence (>95% of the immunized animals) and a synchronized clinical course, with disease onset at day 9 after immunization, a peak of clinical symptoms between days 12 and 20, and a slow resolution from day 21 onwards. We asked whether immunization with the five peptides identified by our screening and with the peptide identified by Iwanami and colleagues [30] also induced arthritis. In several independent experiments, which are summarized in Table 1, immunization with all six G6PI peptides induced arthritis – albeit of different incidence and severity. Immunization with G6PI_65–79_, G6PI_497–511 _and G6PI_517–531 _induced arthritis with varying and comparatively low incidence. Duration of arthritis was short and clinical symptoms were only mild (Table 1). In contrast, immunization with G6PI_85–99_, G6PI_325–339 _and G6PI_469–483 _reproducibly induced arthritis with an incidence between 79 and 100%. Onset of arthritis was delayed, the clinical scores were significantly lower (*P *< 0.05 for G6PI-immunized vs. all peptide immunized groups), and the disease was of shorter duration in the peptide-immunized mice compared with the mice immunized with full-length G6PI (Figure 2a).

**Table 1:** Summary of immunization experiments

Peptide	Arthritic mice per experiment	Cumulative incidence (%)
G6PI_65–79_	3/5	60
	5/5	
	1/5	
G6PI_85–99_	5/5	79
	8/10	
	5/5	
	4/8	
	4/5	
	5/5	
	3/5	
G6PI_325–339_	5/5	100
	4/4	
G6PI_469–483_	5/5	95
	7/8	
	5/5	
	5/5	
G6PI_497–511_	3/5	53
	5/5	
	0/5	
G6PI_517–531_	1/5	40
	5/5	
	0/5	

Histopathological analysis revealed typical signs of arthritis in both peptide-immunized and protein-immunized mice (Figure 2b). Confirming our earlier findings, arthritis was almost exclusively localized to the phalangeal and tarsal joints, whereas the knee joints were rarely and only mildly affected. In most animals, the legs were symmetrically involved, and there was no predilection for the front or the hind limbs.

The inflammation involved the synovial fluid and synovial membrane (Figure 2b, upper row, encircled areas) and extended into the adjacent structures, comprising tendons and tendon sheaths, ligaments and muscles (Figure 2b, upper row, stars), and in the most severe cases it also affected the subcutaneous tissue and skin.

At day 12 the signs of acute and chronic inflammation were similarly developed, with a slight predominance for features of acute arthritis. In the joints of all animals, infiltrating neutrophilic granulocytes could be detected – in the more severe cases with formation of abscesses (Figure 2b, left column) and purulent joint effusion, and with fibrin exudation (Figure 2b, lower row, long arrows).

In contrast, cell types typical of chronic inflammation like lymphocytes, plasma cells and macrophages were found to a lesser extent (Figure 2b, lower row, arrowheads). No formation of multinucleated giant cells and no granulomas could be observed. A thickening of the synovial lining and a dense proliferation of fibroblasts were observed (Figure 2b, lower row, arrowheads), however, comparable with the pannus formation in human rheumatoid arthritis.

Taken together, our data show that three of the peptides were robustly arthritogenic, whereas immunization with the other three peptides resulted only in a slight arthritis induction. We therefore restricted the following analyses of the T-cell and B-cell responses to the arthritogenic peptides G6PI_85–99_, G6PI_325–339 _and G6PI_469–483_.

### *Ex vivo *cytokine production after immunization with G6PI or arthritogenic peptides

To identify G6PI-specific or peptide-specific T cells we used intracellular staining of CD154 after a brief restimulation of *ex vivo *isolated draining lymph node cells with full-length G6PI or the respective peptide. CD154 expression is strictly dependent on T-cell receptor engagement, and therefore expression of CD154 identifies antigen-specific cells in *ex vivo *stimulation assays [28,29].

**Figure 3:**
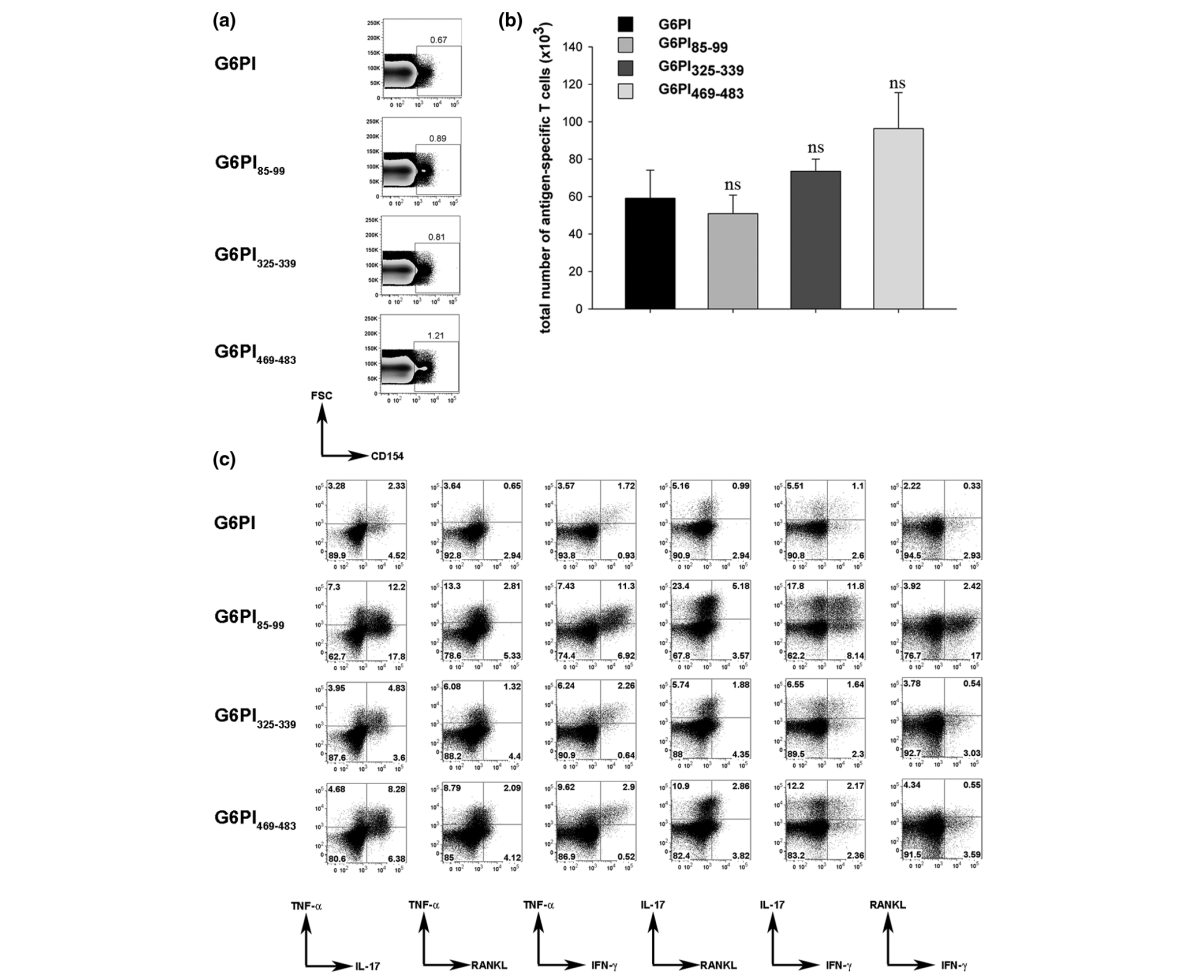
T-cell response after immunization with glucose-6-phosphate isomerase or arthritogenic peptides. DBA/1 mice were immunized with 400 μg glucose-6-phosphate-isomerase (G6PI), 50 μg G6PI_85–99_, 50 μg G6PI_325–339 _or 50 μg G6PI_469–483 _in complete Freund's adjuvant. Mice were sacrificed on day 12 after immunization and single-cell suspensions from draining lymph nodes were prepared. Cells were stimulated with same antigen as used for immunization (20 μg/ml G6PI or 5 μg/ml peptide). **(a) **Antigen-specific CD4^+ ^cells were identified by CD154 expression as described in Materials and methods. Numbers above the gates indicate the percentage of CD154^+ ^cells. **(b) **Total numbers of antigen-specific CD4^+ ^cells were calculated by multiplication of the total number of lymph node cells with the frequency of CD4^+^CD154^+ ^cells. **(c) **Intracellular staining for TNFα, IL-17, RANKL and IFNγ. Dot plots show the expression of these cytokines in antigen-specific T cells (gated on CD4^+^CD154^+ ^cells). Numbers in quadrants indicate the percentage of cells producing the respective cytokines within the antigen-specific T-cell compartment. Data depicted in (a) and (c) are from concatenated data files from five individual mice per group and represent all mice in the respective group. This experiment was performed at least two times. FSC, forward scatter; ns, nonsignificant; *P *> 0.05 between G6PI-immunized vs. peptide-immunized mice.

We first analyzed the total number of CD154^+^CD4^+ ^cells from the draining lymph nodes at day 12 after immunization, and found no statistically significant difference between G6PI-immunized or peptide-immunized mice (Figure 3a, b). Next we examined the cytokine production of the CD154^+ ^antigen-specific T cells. As shown in Figure 3c, the most abundant cytokine in all groups was IL-17, followed by TNFα and RANKL or IFNγ. The frequency of antigen-specific IL-2-producing, IL-4-producing or IL-6-producing T cells was extremely low, and IL-10-producing Th cells were never detected in G6PI-immunized mice (data not shown).

Compared with mice immunized with full-length G6PI, peptide-immunized mice showed a more prominent cytokine response. It is important to note here that the T-cell response develops much faster in the animals immunized with full-length G6PI, with a maximum response at day 9, compared with the peptide-immunized mice, in which the peak response is at day 12 after immunization ([21] and data not shown).

### G6PI-specific immunoglobulins after immunization with G6PI or immunodominant peptides

**Figure 4:**
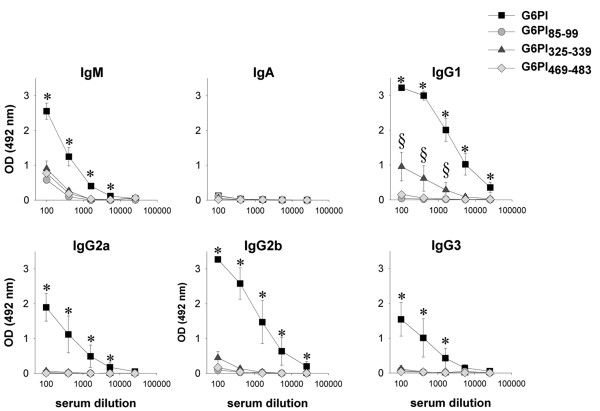
Analysis of glucose-6-phosphate-specific immunoglobulin production. DBA/1 mice were immunized with either 400 μg glucose-6-phosphate (G6PI), 50 μg G6PI_85–99_, 50 μg G6PI_325–339_or 50 μg G6PI_469–483 _in complete Freund's adjuvant. Mice were sacrificed on day 12 after immunization and titers of G6PI-specific immunoglobulins of the indicated isotypes were measured by ELISA. Data are representative of at least two different experiments (n = 4 per group; *P < 0.05 for G6PI-immunized vs. peptide-immunized mice; ^§^*P *< 0.05 for G6PI_325–339 _vs. G6PI_85–99 _or G6PI_469–483_). OD, optical density.

Since we have previously shown that B-cell responses are important for the pathogenesis of G6PI-induced arthritis [21,22], sera of the mice were tested for the presence of G6PI-specific immunoglobulins. As expected, immunization with full-length G6PI resulted in high titers of all isotypes except IgA (Figure 4). In mice immunized with G6PI_85–99 _or G6PI_469–483 _we detected only G6PI-specific IgM, albeit at much lower concentrations than in G6PI-immunized mice. We additionally detected small amounts of G6PI-specific IgG_1 _and IgG_2b _in the sera of animals immunized with G6PI_325–339 _(Figure 4).

## Discussion

In the present article we describe the identification of two novel peptides derived from human G6PI, which are arthritogenic in DBA/1 mice. In addition, we confirm the report by Iwanami and colleagues identifying G6PI_325–339 _as an arthritogenic peptide [30]. The peptide sequence of G6PI_469–483 _is identical in man and mouse. G6PI-induced arthritis is therefore currently the only mouse model in which arthritis can be induced by immunization with a peptide derived from a self-antigen.

Our screen identified at least six G6PI peptides that are immunodominant for I-A^q^-restricted T-cell responses. The T-cell response towards the ubiquitously expressed autoantigen G6PI is therefore not focused on one dominant epitope. Instead, at least three peptide–epitopes derived from G6PI are immunodominant and arthritogenic in DBA/1 mice. There is precedence for several immunodominant antigens within one protein for T-cell responses restricted towards one particular MHC molecule [31]. For hen egg lysozyme, which consists of only 129 amino acids, there are at least six dominant peptide epitopes for I-A^k^-restricted T-cell responses alone [32].

The different number of peptides identified by Iwanami and colleagues [30] and in the present report reflects the fundamentally different approaches to identifying immunodominant G6PI epitopes. Iwanami and colleagues examined known sequences of I-A^q^-restricted T-cell epitopes and deduced a possible I-A^q ^binding motif from these sequences. Peptides derived from G6PI that fit this possible I-A^q ^binding motif were then synthesized and tested. These peptides covered 399/558 (71.5%) amino acid residues of the human GPI protein. Consequently, as acknowledged by Iwanami and colleagues [30], this approach carries the risk of missing relevant peptide epitopes. Moreover, several groups including ours have shown previously that several peptides that do not fit the consensus sequence for peptides binding to a given MHC molecule can activate T cells restricted to that MHC molecule very efficiently [33-36]. We therefore took an unbiased approach. To identify the immunodominant epitopes we used a set of 15mer peptides overlapping by 11 amino acids that span the whole sequence of human G6PI. Neither G6PI_85–99 _nor G6PI_469–483 _were predicted by the algorithm used by Iwanami and colleagues [30], and neither of these two peptides fits the binding motif for I-A^q ^that has been suggested by Holm and colleagues based on their analysis of 24 I-A^q^-binding peptides [37] – again supporting the use of unbiased approaches to epitope identification.

Whereas these considerations explain how Iwanami and colleagues might have missed G6PI_85–99 _and G6PI_469–483_, the fact that our analyses did not identify G6PI_325–339 _still needs explanation. In our library G6PI_325–339 _was peptide 82. Therefore it was included in Pools 10 and 19. While peptide Pool 10 scored positive in all three experiments, Pool 19 scored positive only once – therefore peptide 82, which occurred in these two pools, was initially not synthesized for further analyses. There are several possible reasons for the altogether weak proliferation data obtained from Pool 19, including the possibility that agonist and weak antagonist peptides contained within such a pool could cancel out one another [38]. Nevertheless, unbiased large peptide libraries have been used very successfully to identify T-cell epitopes [33-36].

Another difference between our findings and those reported by Iwanami and colleagues [30] is the kinetic and clinical severity of arthritis induced by peptide as compared with arthritis induced by G6PI protein. In our hands, peptide-induced arthritis occurs somewhat delayed and with lower incidence and clinical severity than G6PI-induced arthritis. Iwanimi and colleagues report no difference in arthritis severity and onset between peptide-immunized and protein-immunized mice. This difference is most probably due to the fact that our immunization protocol uses antigen in CFA subcutaneously for both peptide and protein immunization, whereas Iwanami and colleagues use intradermal injection of peptide in CFA followed by two injections of pertussis toxin intraperitoneally at days 0 and 2 relative to immunization [30]. In fact, Iwanami and colleagues report on lower incidence and severity of arthritis when they omit pertussis toxin. Both groups observe substantially lower G6PI-specific antibody titers in the peptide-immunized mice as compared with G6PI-immunized mice. Given we have shown earlier that FcγR is critical for arthritis development [21], it seems likely that the low antibody concentrations detectable in the serum are sufficient to contribute to the milder form of arthritis induced upon peptide immunization. Moreover, it has been reported that pathogenic antibodies against G6PI attach to cartilage and therefore accumulate in the joints [39,40].

The number of antigen-specific Th cells was similar in the draining lymph nodes of peptide-immunized or protein-immunized mice. We used CD154 expression to identify G6PI-specific Th cells. CD154 expression is rapidly upregulated upon T-cell receptor signaling, and CD154 expression has been shown to be a sensitive and specific marker to identify T cells specific for a defined antigen [28,29,41-43]. To examine the G6PI-specific cytokine responses, we determined the frequency of cytokine producers among the CD4^+^CD154^+ ^Th cells upon *in vitro *culture with antigen. Perhaps unexpectedly, the frequency of cytokine producers among the antigen-specific Th cells was higher in the peptide-immunized mice than in the protein-immunized mice. In addition there were also differences among the mice immunized with different peptides. For example, the highest frequency of IL-17-producing CD4^+^CD154^+ ^cells was found in the draining lymph nodes of mice immunized with G6PI_85–99_. Caution is warranted in interpreting fine quantitative differences among the different groups. Several confounding parameters, including different antigen-processing requirements for full-length G6PI protein or peptides, dissimilar kinetics of the responses and the differing solubility of individual G6PI peptides, make it difficult to compare quantitatively the frequency of cytokine-producing Th cells in response to G6PI or peptides in the different groups of mice.

Our data add RANKL to the list of cytokines that are prominently produced by G6PI-specific Th cells. IL-6 is a therapeutic target in juvenile idiopathic arthritis and rheumatoid arthritis in humans [44]. It is also produced upon polyclonal stimulation of T cells from G6PI-immunized mice [21]. Matsumoto and coworkers recently found that IL-6 was relevant for the pathogenesis of G6PI-induced arthritis [24,25]. Interestingly, IL-6 is not produced by G6PI-specific Th cells directly *ex vivo *upon culture with either G6PI or G6PI peptides. Cells other than the G6PI-specific Th cells must therefore produce the pathogenetically relevant IL-6 in G6PI-induced arthritis. B cells, which are required for the pathogenesis of G6PI-induced arthritis [22] and also for rheumatoid arthritis [45,46], are potent producers of IL-6. It shall be interesting to determine which cell population provides the pathogenic IL-6 in G6PI as well as in rheumatoid arthritis. Except for the different kinetics, the pattern of Th-cell cytokine production is very similar in G6PI-immunized and G6PI_85–99_-immunized mice. Compared with these two groups, the G6PI_469–483_-immunized mice harbor much fewer CD4^+^CD154^+ ^cytokine producers. This does not seem to correlate with the incidence and severity of arthritis, which is very similar in G6PI_85–99_-immunized mice and G6PI_469–483_-immunized mice.

## Conclusions

In the present article we describe the identification of six immunodominant G6PI peptides that induce T-cell responses in DBA/1 mice. Immunization with three of these peptides induces peripheral symmetric polyarthritis with high incidence. One of the peptides (G6PI_469–483_) is an autoantigen. We have therefore described for the first time arthritis in mice induced by immunization with a self-peptide.

## Abbreviations

APC: antigen-presenting cells; CFA: complete Freund's adjuvant; ELISA: enzyme-linked immunosorbent assay; FCS: fetal calf serum; G6PI: glucose-6-phosphate-isomerase; H & E: hematoxylin and eosin; IFN: interferon; IL: interleukin; mAb: monoclonal antibody; RANKL: receptor activator of NFκβ ligand; Th: T-helper.

## Competing interests

The authors declare that they have no competing interests.

## Authors' contributions

LB and OF participated in the *in vivo *studies, the T-cell assays and the ELISA studies, and drafted parts of the manuscript. LM performed the histopathological analyses. CL and RV prepared the peptide libraries, the peptides and participated in designing the peptide pools. TK conceived of the study and participated in its design and coordination, and wrote the manuscript. All authors read and approved the final manuscript.
